# Phenotypic modifications in ovarian cancer stem cells following Paclitaxel treatment

**DOI:** 10.1002/cam4.115

**Published:** 2013-08-27

**Authors:** Vinicius Craveiro, Yang Yang-Hartwich, Jennie C Holmberg, Natalia J Sumi, John Pizzonia, Brian Griffin, Sabrina K Gill, Dan-Arin Silasi, Masoud Azodi, Thomas Rutherford, Ayesha B Alvero, Gil Mor

**Affiliations:** 1 Department of Obstetrics, Gynecology and Reproductive Sciences, Reproductive Immunology Unit, Yale University School of MedicineNew Haven, Connecticut; 2 Bruker CorporationBillerica, Massachusetts

**Keywords:** EMT, ovarian cancer stem cells, recurrence, slug

## Abstract

Epithelial ovarian cancer (EOC) is the most lethal gynecologic malignancy. Despite initial responsiveness, 80% of EOC patients recur and present with chemoresistant and a more aggressive disease. This suggests an underlying biology that results in a modified recurrent disease, which is distinct from the primary tumor. Unfortunately, the management of recurrent EOC is similar to primary disease and does not parallel the molecular changes that may have occurred during the process of rebuilding the tumor. We describe the characterization of unique in vitro and in vivo ovarian cancer models to study the process of recurrence. The in vitro model consists of GFP+/CD44+/MyD88+ EOC stem cells and mCherry+/CD44−/MyD88− EOC cells. The in vivo model consists of mCherry+/CD44+/MyD88+ EOC cells injected intraperitoneally. Animals received four doses of Paclitaxel and response to treatment was monitored by in vivo imaging. Phenotype of primary and recurrent disease was characterized by quantitative polymerase chain reaction (qPCR) and Western blot analysis. Using the in vivo and in vitro models, we confirmed that chemotherapy enriched for CD44+/MyD88+ EOC stem cells. However, we observed that the surviving CD44+/MyD88+ EOC stem cells acquire a more aggressive phenotype characterized by chemoresistance and migratory potential. Our results highlight the mechanisms that may explain the phenotypic heterogeneity of recurrent EOC and emphasize the significant plasticity of ovarian cancer stem cells. The significance of our findings is the possibility of developing new venues to target the surviving CD44+/MyD88+ EOC stem cells as part of maintenance therapy and therefore preventing recurrence and metastasis, which are the main causes of mortality in patients with ovarian cancer.

## Introduction

Epithelial ovarian cancer (EOC) accounts for more than 90% of all ovarian neoplasms and remains the leading cause of gynecologic cancer deaths [1, 2]. In the United States, more than 20,000 new cases are diagnosed annually and ∼14,000 women succumb to the disease each year. Patient survival rate depends on the stage of the disease at time of diagnosis. Approximately 20% of newly diagnosed cases are classified as Stage I and these patients have a 10-year survival rate of about 73% [3]. Unfortunately, almost 85% of patients are diagnosed with Stages III–IV disease and in these patients, the 10-year survival rate drops to 5–20% [3] and mortality is almost always associated with recurrent disease.

All newly diagnosed EOC patients undergo surgery, which is required for diagnosis and staging, in addition to providing cytoreduction. Adjuvant chemotherapy is given to most patients and usually consists of the combination of carboplatin and Paclitaxel. Although most patients achieve complete remission after initial surgery and chemotherapy, more than 80% of patients will present with recurrent disease [4]. Patients with recurrent EOC mostly present with carcinomatosis, which is not amenable to surgical debulking, and they usually develop resistance to most of the currently available antitumor agents [4]. The key biological processes leading to the formation of very aggressive and highly metastatic recurrent disease is not clearly understood.

Cancer cells that comprise tumors are heterogeneous both morphologically and functionally. Individual tumors show distinct subareas of proliferation and differentiation, specifically epithelial–mesenchymal transition (EMT). Contrary to the stochastic model of cancer (clonal expansion), the cancer stem cell (CSC) model holds that tumors are hierarchically organized and only some cells have the capacity to indefinitely self-renew and sustain tumor growth [5–7]. It is thought that CSC are able to survive conventional chemotherapies, which usually target faster dividing cells and give rise to recurrent tumors that are more resistant and more aggressive [5, 6, 8–10]. However, we have limited understanding on the phenotype and biological characteristics of the surviving CSC. Do they maintain the same characteristics as the original tumor-initiating cells? And in addition, do recurrent tumors maintain characteristics similar to the primary tumor?

One of the major hurdles in identifying the specific phenotype of ovarian CSC is the heterogeneity of the disease [11]. Ovarian cancer can be classified into multiple types (serous, endometrioid, clear cell, and mucinous), with each type having widely different clinicopathologic properties [12–15]. It is therefore possible that each of these types of ovarian cancer has its own unique ovarian CSC phenotype. Thus, it is not surprising that stemness properties have been reported in ovarian cancer cells that have been isolated using a variety of cell surface markers, such as CD44, CD133, and CD24 [8, 16–19]. Each of these ovarian cancer cell phenotypes may represent either a hierarchy of CSC or an entirely different population of CSC for that particular ovarian cancer histotype. In spite of the lack of consensus about the markers for ovarian CSC, several groups including our own have reported the role of CD44+ ovarian cancer cells in tumor initiation and chemoresistance [8, 20–24] – a role that has been ascribed to CSC. Thus irrespective of its hierarchy, the presence of CD44+ ovarian cancer cells has been correlated with processes observed during ovarian cancer recurrence, such as chemoresistance, tumor repair, and carcinomatosis.

Our group has elaborated on the molecular phenotype and cellular properties of these cells. In addition to having tumor-initiating properties, the CD44+/MyD88+ subtype of EOC cells express multiple pluripotency-associated genes, can differentiate into vascular tumor progenitors and, in addition, undergo EMT to yield migratory mesenchymal CSCs [8, 9, 25–27]. However, probably one of the most fundamental properties of these cells is the inherent resistance to currently available chemotherapeutic agents. In fact, although there is no consensus in the hierarchical place of CD44+ ovarian cancer cells in terms of its stemness property, there is a consensus that CD44+ ovarian cancer cells represent a more chemoresistant phenotype [8, 21–24].

In this study, we describe the characterization of unique in vitro and in vivo ovarian cancer models to study the process of recurrence. Using these models we confirmed that chemotherapy enriched for CD44+/MyD88+ EOC stem cells. However, we observed that the surviving CD44+/MyD88+ EOC stem cells acquire additional characteristics not present prior treatment. Our findings suggest that chemotherapy does not only enrich for putative EOC stem cells, but also that treatment can induce specific phenotypic modifications in the surviving EOC stem cells. These findings are important for evaluating the therapeutic approach for recurrent disease.

## Material and Methods

### Cell cultures and conditions

We have established six clones of CD44+/MyD88+ EOC stem cells and their derived daughter cells (CD44−/MyD88− EOC cells), purified from patients diagnosed with serous EOC, as previously described [8, 25, 26]. For the in vitro and in vivo model we mainly described the studies using clone OCSC1 (CD44+/MyD88+ EOC cells). OCC1 (CD44−/MyD88− EOC cells) are derived from OCSC 1 by in vitro differentiation through serial low concentration passaging as previously described [28] and have similar characteristics as CD44−/MyD88− EOC cells isolated from primary tumors. We previously demonstrated that OCSC1 can differentiate into OCC1 and that OCC1 is not a cell contaminant that can overtake OCSC1 in culture [28]. Purity of the cultures was tested before each experiment by measuring the levels of CD44 and MyD88. All sample collections described in this study were performed with patient consent and approved by the Human Investigations Committee of Yale University School of Medicine.

### Determination of cell growth and morphology

Effect of Paclitaxel treatment on cellular morphology and proliferation was assessed using Incucyte (Essen Instruments, Ann Arbor, MI). Proliferation was measured through quantitative kinetic processing metrics derived from time-lapse image acquisition and presented as percentage of culture confluence over time.

### Generation of fluorescently labeled EOC cells using lentivirus

GFP-labeled OCSC1 and mCherry-labeled OCC1 cells were established by infecting cells with lentivirus expressing the fluorescent proteins. Lentivirus was produced using a polyethylenimine (PEI) protocol [29]. Briefly, HEK 293T cells were seeded in a 10 cm plate at a density of 5 × 10^6^ cells. When confluence reached 80% transfection was performed by adding a mixture of 10 μg plasmid DNA (5:2:3, psPAX: pMD2G: FUGW or FUCW) and 30 μg PEI. Virus was harvested after 48 h and concentrated by ultracentrifugation. Viral infection was done in suspension using 10^6^ cancer cells in a sterile microfuge with concentrated viral particles and 1 mL of fresh RPMI 1640 media. The cancer cells were incubated 1–2 h at 37°C, shaking intermittently. After incubation the cancer cells were transferred to a 75 cm^2^ tissue culture flask by adding 6 mL of fresh media. The next day, the cancer cells were inspected for fluorescence by microscopy and flow cytometry. The viral media was removed and replaced with fresh RPMI to allow the cancer cells to recover.

### Establishment of Slug-overexpressing cell line

Slug-overexpressing cell line was established by retroviral infection. Platinum-A retroviral packaging cell line (Cell Biolabs, Inc., San Diego, CA) was used to produce retrovirus. Platinum-A retroviral packaging cells were seeded in a 10 cm plate at a density of 5 × 10^6^ cells. When the confluence reached 80% transfection was performed by adding a mixture of 10 μg plasmid DNA (pBABE-puro-slug or pBABE-puro) and 30 μg PEI to Platinum-A cells. Conditioned medium, which contained retrovirus was harvested at 24 h and then added to the target cells. After 24 h incubation, puromycin was added to the target cells medium to select the infected cells. Slug expression in puromycin-selected cells was tested by western blot to confirm overexpression.

### Caspase activity assay

Protein was extracted and measured as previously described [21, 30]. Activity of caspase 3/7 was quantified using Caspase Glo 3/7 (Promega, Madison, WI) according to manufacturer's instructions.

### Protein preparation, SDS-PAGE, and Western blots

Total cellular protein was extracted as previously described [21, 30]. Sodiumdodecyl sulfate polyacrylamide gel electrophoresis (SDS-PAGE) and Western blots were performed using 20 μg of total protein lysate as previously described. Antibodies used were rabbit anti-Klf4 (Abcam, Cambridge, MA), rabbit anti-Slug (Cell Signaling Technology, Danvers, MA), rabbit anti-Oct4 (Cell Signaling), rabbit anti-Nanog (Cell Signaling), and rabbit antiactin (Sigma Aldrich, St. Louis, MI). Beta-actin antibody was purchased from Sungene Biotech, (Tianjin, China) clone KM9001.

### Quantitative PCR

Total cellular RNA was isolated using RNeasy kit and 1 μg of RNA was reverse transcribed to cDNA using Verso cDNA kit (both kits were used according to manufacturers' instructions). cDNA at 1:10 dilution was used for each polymerase chain reaction (PCR) with appropriate primers and using KAPA SYBR FAST Universal 2× qPCR kits (Kapa Biosystems, Woburn, MA). Primer sequences are as follows: *ALDH* (5′-tgcgctactgtgcaggttggg-3′ and 5′-ccacagctcagtgcaggccc-3′)*; CD44* (5′-gacagcacagacagaatc-3′ and 5′-gtgagtgtccatctgattc-3′); *OCT4* (5′-gatgtggtccgagtgtggttct-3′ and 5′-tgtgcatagtcgctgcttgat-3′); *KLF4* (5′-tctcaaggcacacctgcgaa-3′ and 5′-tagtgcctggtcagttcatc-3′); *MYD88* (5′-acgtgctgctggagctg-3′ and 5′-gatcagtcgcttctgatg-3′)*; NANOG* (5′-gcagaaggcctcagcaccta-3′ and 5′-aggttcccagtcgggttca-3′)*; SNAI2* (5′-tgacctgtctgcaaatgctc-3′ and 5′-cagaccctggttgcttcaa-3′); *TWIST1* (5′-gtcatggccaacgtgcggga-3′ and 5′-gccgccagcttgagggtctg-3′); *VIM* (5′-attccactttgcgttcaagg-3′ and 5′-cttcagagagaggaagccga-3′); and *ZEB1* (5′-tgcagtttgtcttcatcatctg-3′ and 5′-ccaggtgtaagcgcagaaa-3′). All PCR reactions were performed on CFX96- Real-Time System (Bio-Rad, Hercules, CA) in triplicate and validated by the presence of a single peak in the melt curve analysis. Changes in gene expression were calculated relative to *ACTB* (5′-ttgccgacaggatgcagaagga-3′ and 5′-aggtggacagcgaggccaggat-3′) using the 2^−ΔΔCt^ method.

### Flow cytometry analysis

Extracellular expression of CD44 was determined by staining cells with rat anti-human/mouse CD44-FITC (ebioscience, San Diego, CA) according to manufacturer's instructions. Data were acquired using BD FACS Calibur (BD Bioscience, San Jose, CA) and analyzed using CellQuest (BD Bioscience).

### Immunohistochemistry

Immunohistochemistry was performed as previously described [9] using rabbit antivimentin (cell signaling).

### Generation of in vivo models

The Yale University Institutional Animal Care and Use Committee approved all in vivo studies described. For the tumor implant model, a s.c. tumor implant was established in athymic nude mice as previously described [9]. Briefly, following a lateral skin incision a 5 mm^3^ tumor fragment from a patient with recurrent EOC was introduced subcutaneously. The skin was sealed and tumor growth monitored weekly. For the i.p. recurrence model, OCSC1 was injected i.p. in an athymic nude mouse and the resulting F2 tumor dissociated and transfected with mCherry fluorescent protein. 7 × 10^6^ mCherry+ OCSC1-F2 cells were injected i.p. per mouse to establish i.p. carcinomatosis. Paclitaxel was given i.p. at 20 mg/kg q3d and Cisplatin was given i.p. at 5 mg/kg once a week. Tumor growth was monitored q3d by imaging using in vivo Imaging system FX PRO (Bruker Corp., Billerica, MA). Tumor load was monitored daily with caliper measurements of the abdominal circumference.

### Statistical analysis

Data are presented as the mean ± standard deviation (SD). A Student's *t* test was used to calculate the *P* values. *P* < 0.05 is considered significant.

## Results

### Differential response of ovarian cancer cell subtypes to Paclitaxel

The in vitro evaluation of drug efficacy in cancer cells is usually done using cell viability assays that determine the number of viable cells at the end of the experiment. The majority of these assays are terminal and do not evaluate the outcome in surviving cells. In this study, we evaluated the effect of Paclitaxel on ovarian cancer cells by monitoring their response in real time and determining the consequence of exposure to its molecular phenotype. We used two subtypes of ovarian cancer cells based on their expression of CD44 and MyD88. We have described the characterization of these cell clones in several publications [8, 31, 32]. The clone OCSC1 used in this study is 100% CD44+, MyD88+, and ALDH+ (CD44+/MyD88+) [28]. These cells can undergo differentiation in vitro and in vivo to give origin to OCC1, which are negative for CD44, MD88, and ALDH (CD44−/MyD88− EOC cells) [9]. In our previous reports, we have shown that OCSC1 can rebuild the original tumor in mice (tumor-initiation potential), express stemness-associated markers, can serve as tumor vascular progenitors (pleuripotency), and are more chemoresistant [8, 9, 21, 25–27]. In contrast, OCC1 have lost the capacity to initiate tumors, and are more chemosensitive [31].

**Figure 1:**
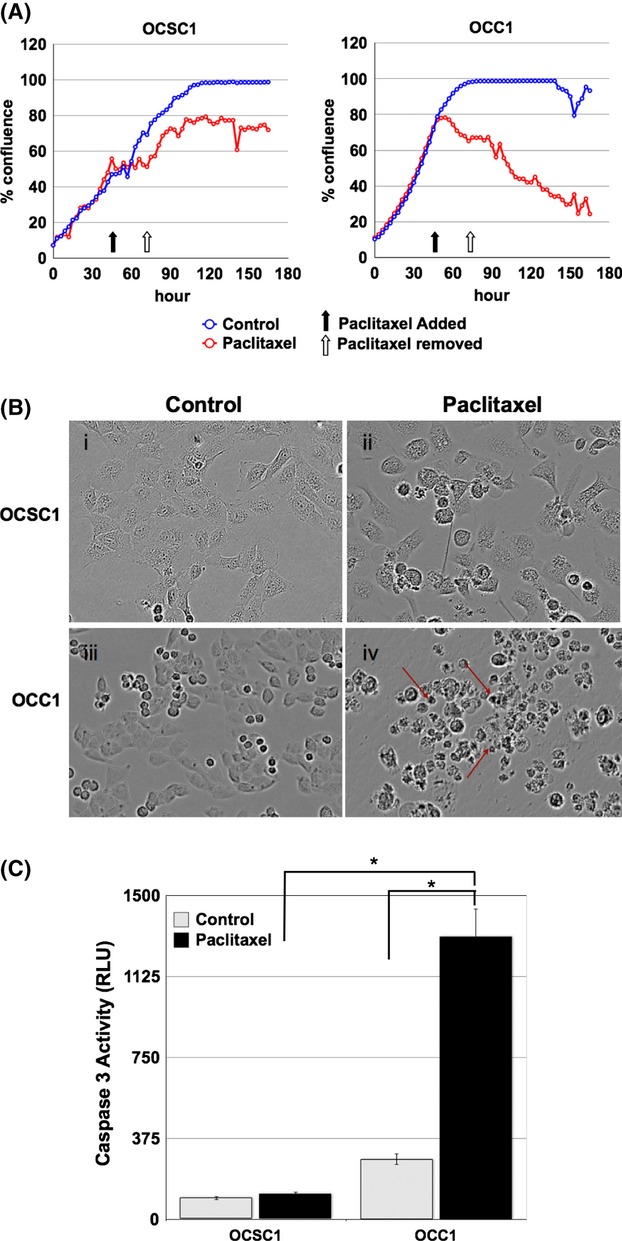
Paclitaxel induces apoptosis only in CD44−/MyD88− OCC1 cells. Cells were treated with 0.2 μmol/L Paclitaxel for 48 h and allowed to recover for another 72 h. (A) Culture confluence was monitored by the Incucyte live-imaging system. Black arrow indicates the time of treatment initiation. White arrow indicates time of wash out of the treatment. Data is representative of three independent experiments. (B) Morphology assessed at 48 h posttreatment. Note the presence of apoptotic cells in OCC1 treated with Paclitaxel (red arrows). (C) Caspase-3 activity was measured 48 h posttreatment. Note the significant increase in caspase-3 activity in OCC1 following Paclitaxel treatment. *P < 0.005.

Our first objective was to determine the capacity of OCSC1 and OCC1 to repopulate the cell culture following therapy. Paclitaxel (0.2 μmol/L) was added to OCSC1 and OCC1, maintained in culture for 48 h, subsequently replaced with regular growth medium and cells monitored for an additional 3 days. This dose was chosen because previous studies have shown this dose as the GI_50_ for classical ovarian cancer cell lines [32]. As shown in Figure 1A, the growth curve of OCSC1 plateaued upon addition of Paclitaxel, but the cells recuperated and resumed proliferative growth when the compound was removed. In contrast, OCC1 lost viability when Paclitaxel was added and did not recover even when the compound was removed (Fig. 1A, white arrow). Further analysis of the surviving cells showed that a high percentage of OCSC1 maintained their morphological characteristics and adherence to the plate after Paclitaxel treatment (Fig. 1B-i and -ii). In contrast, OCC1 displayed apoptotic morphology (red arrows) (Fig. 1B-iii and -iv). Evaluation of caspase-3 activity confirmed the activation of the apoptosis pathway. We saw high levels of active caspase-3 only in Paclitaxel-treated OCC1 and not in OCSC1 (Fig. 1C). These data confirmed that OCSC1 (CD44+/MyD88+ cells) are resistant to Paclitaxel and maintain their capacity to replicate and grow after treatment.

**Figure 2:**
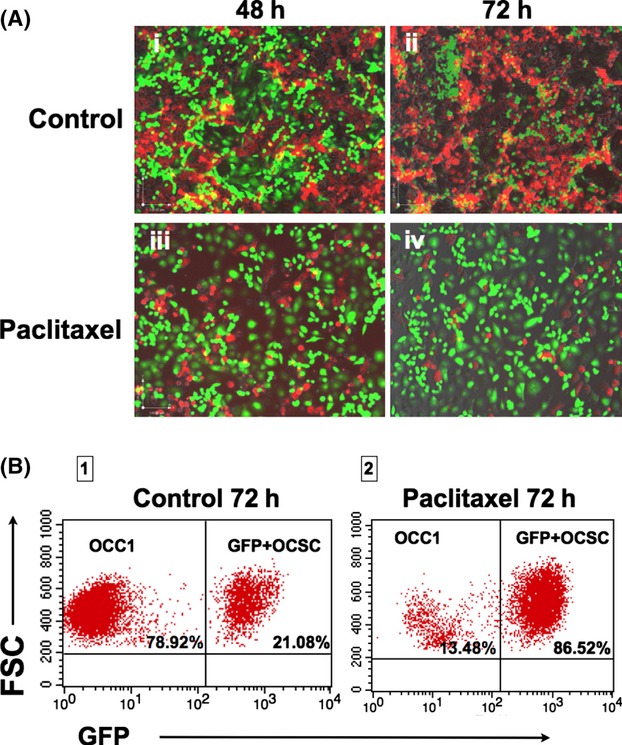
Paclitaxel enriches for CD44+/MyD88+ OCSC1 cells. Cocultures of GFP+ OCSC1 and mCherry+ OCC1 were treated with 0.2 μmol/L Paclitaxel for 48 h and allowed to recover for another 72 h. (A) Fluorescence was determined by fluorescence microscopy. (i–ii) Note the overgrowth of mCherry+ OCC1 (red) in relation to GFP+ OCSC1 (green) in the control group; (iii–iv) Mainly GFP+ ovarian cancer stem cell (OCSC)1 (green) are observed in cultures treated with Paclitaxel. (B) Enrichment of GFP+ OCSC1 cells following Paclitaxel treatment determined by flow cytometry. Data are representative of five independent experiments.

Ovarian cancer tumors are heterogeneous and in our previous reports we have shown that both subtypes of cells (CD44+/MyD88+ and CD44−/MyD88−) are present in the tumors [28]. To determine whether heterogeneity can affect the response to Paclitaxel, we developed an in vitro coculture model that mimics the cellular heterogeneity observed in patients' tumors. This in vitro coculture system consists of 50% GFP-labeled OCSC1 (CD44+/MyD88+) and 50% mCherry-labeled OCC1 (CD44−/MyD88−) cells. The cocultures were treated with 0.2 μmol/L Paclitaxel and after 48 h, Paclitaxel was washed-off and replaced with regular growth media. The cocultures were further monitored for another 72 h. In control cocultures, mCherry+ OCC1 were observed to comprise the majority of cells by 72 h (Fig. 2A-ii). This was expected as OCC1 have a faster growth rate compared to OCSC1. In contrast, Paclitaxel-treated cocultures are composed mainly of GFP+ OCSC1 (Fig. 2A-iii and -iv). The enrichment of OCSC1 by Paclitaxel is further confirmed by flow cytometry. Control cocultures contained 21.08% GFP+ OCSC1 and 78.92% OCC1 (Fig. 2B-1). In contrast, Paclitaxel-treated cocultures contained 86.52% GFP+ OCSC1 and 13.48% OCC1 (Fig. 2B-2). Taken together these results confirm our previous reports that the cytotoxic effect of Paclitaxel is limited only against CD44−/MyD88− EOC cells and the treatment enriched for CD44+/MyD88+ cells [8, 21, 31].

### CD44+/MyD88+ EOC cells maintain proliferative potential but are phenotypically modified postpaclitaxel treatment

**Figure 3:**
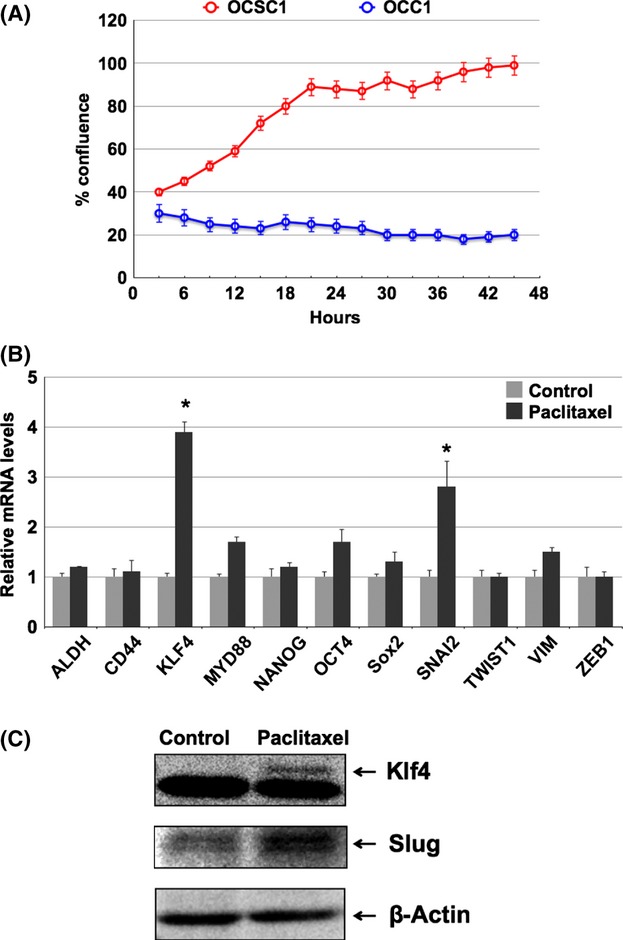
Paclitaxel upregulates stemness- and mesenchymal-associated markers in the surviving CD44+/MyD88+ OCSC1 cells. (A) Cells were treated with 0.2 μmol/L Paclitaxel for 24 h and proliferative potential determined by replating the cells. Only OCSC1 are able to repopulate the culture following Paclitaxel treatment. (B) Expression of mRNA stemness- and mesenchymal-associated genes in the surviving OCSC1 following Paclitaxel treatment compared to the nontreated OCSC1 cells. Expression levels were determined by qPCR. *P < 0.05 compared to Control. (C) Western blot analysis for the expression of Klf4 and Slug in the surviving OCSC1 following Paclitaxel treatment compared to the nontreated OCSC1 cells (Control).

As OCSC1 survives Paclitaxel treatment our next objective was to characterize the consequence of Paclitaxel exposure in these cells. First, we evaluated whether the cells that survived treatment maintained their proliferative potential. Thus, OCSC1 and OCC1 were treated with 0.2 μmol/L Paclitaxel for 24 h, allowed to recover in growth media for another 24 h, and cells trypsinized and transferred to new culture plates. As shown in Figure 3A, Paclitaxel-treated OCSC1 was able to repopulate the culture, ultimately reaching 100% confluence. As expected, Paclitaxel-treated OCC1 was unable to reestablish a culture. Taken together, these findings demonstrate that not only can OCSC1 survive Paclitaxel, but more importantly these cells can regain proliferative potential at the cessation of treatment.

We then evaluated whether Paclitaxel modified the molecular phenotype of the surviving OCSC1. Thus, we compared control cultures of OCSC1 (not exposed to Paclitaxel) and OCSC1 that survived Paclitaxel treatment. Using qPCR, we tested the expression of a panel of genes classically associated with stemness and mesenchymal properties. With regards to genes associated with stemness the levels of *ALDH1*, *CD44*, *MYD88, NANOG, OCT4,* and *SOX2* were maintained and thus not significantly different, but *KLF4* was significantly upregulated (fourfold) in the OCSC1 that survived Pacltiaxel treatment (Fig. 3B). With regards to genes associated with mesenchymal phenotype, our results showed that whereas *TWIST1, VIM,* and *ZEB1* were either unchanged or slightly upregulated, levels of *SNAI1* (Slug) is significantly higher in the OCSC1 that survived Pacltiaxel treatment (threefold), compared to no treatment control. Upregulation of KLF4 and SLUG were further confirmed at the protein level by Western blot analysis, which showed the induction of KLF4 and Slug in the OCSC1 that survived Pacltiaxel treatment (Fig. 3C). Taken together, these data show that postpaclitaxel treatment, the surviving OCSC1 maintained its stemness phenotype but, more importantly, acquired genes associated with mesenchymal characteristics.

### Phenotypic modifications induced by Paclitaxel in vivo

**Figure 4:**
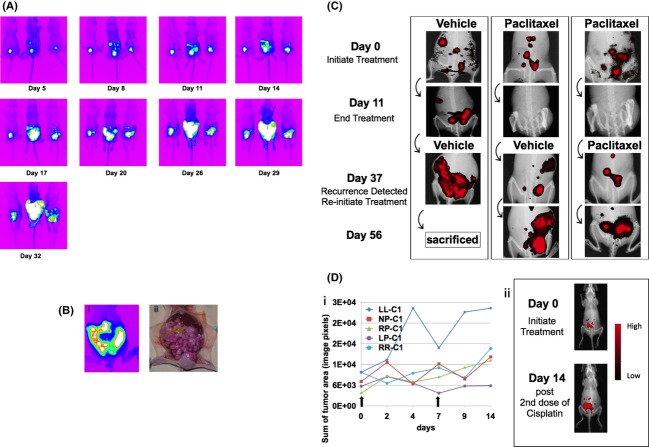
Recurrent i.p. ovarian cancer model. (A) Intraperitoneal tumors were established with mCherry+ ovarian cancer stem cell (OCSC1-F2) as described in the Material and Methods section. Tumor burden was monitored for 32 days. Data shown are representative of five independent experiments (*n* = 10). (B) Correlation between mCherry fluorescent signal obtained from live imaging and actual mouse tumor burden. (i) representative image obtained from in vivo FX system 32 days postinjection of cells; (ii) corresponding photograph of carcinomatosis observed postmortem. (C) Treatment with Paclitaxel was initiated and carried out as described in the text. Note that tumors are undetectable in the treated group after four doses of Paclitaxel (Day 11). Recurrence develops in all mice (Day 37). Recurrent disease was not responsive to the second round of Paclitaxel treatment (Day 56). Data shown are representative of five independent experiments (*n* = 10 animals per group). (D) Treatment with Cisplatin was carried out as described in the text. (i) plot of region of interest (ROI) tumor area from five representative animals treated with Cisplatin; (ii) representative images comparing Control and Cisplatin-treated mouse. Note that in contrast to Paclitaxel, mice progressed with Cisplatin treatment.

To determine if the changes observed in vitro in OCSC1 (maintenance of stemness properties and the acquisition of mesenchymal-associated genes) as a result of Paclitaxel treatment are reproducible in vivo, we established an ovarian cancer xenograft model. Thus, mCherry-labeled OCSC1-F2 cells (with tumor-initiating capacity) were injected i.p. in nude mice and tumor formation was monitored using live in vivo imaging as described in the Material and Methods section [33]. Injection of OCC1 under similar conditions does not form tumors (data not shown and [8]). Once tumor is detected (region of interest [ROI] interior area ∼2000), mice received either vehicle or Paclitaxel (20 mg/kg i.p. q3d). Figure 4A is a representative image of the tumor load observed in vehicle-treated mice through time. In these mice, ROI area can reach >100,000. These mice develop an aggressive intraabdominal carcinomatosis as shown by fluorescent imaging (Fig. 4B-i) and corresponding dissection (Fig. 4B-ii). In mice treated with Paclitaxel, we observed response after the 4th dose of Paclitaxel (Fig. 4C). Response was defined as ROI interior area <2000. At this time, treatment was discontinued and mice were considered disease free. Imaging was further continued to monitor recurrence. Similar to what is observed in ovarian cancer patients, all mice that were considered disease-free after Paclitaxel developed recurrent disease ∼30 days after treatment was terminated (Fig. 4C). As the tumors responded initially to Paclitaxel, we then determined whether recurrent disease is still responsive to the treatment. Thus, once recurrence was observed, mice were randomly assigned to either vehicle or Paclitaxel (20 mg/kg i.p. q3d). Interestingly, none of the mice with recurrent disease responded to the second round of Paclitaxel treatment (Fig. 4B).

We also determined the efficacy of Cisplatin against the i.p. recurrent model. Cisplatin was administered i.p. and given at 5 mg/kg once a week. After the second dose of Cisplatin, however, we observed cachexia with mice losing 20% of their body weight. Treatment was therefore suspended. Analysis of tumor load showed that the mice progressed with treatment (Fig. 4D).

**Figure 5:**
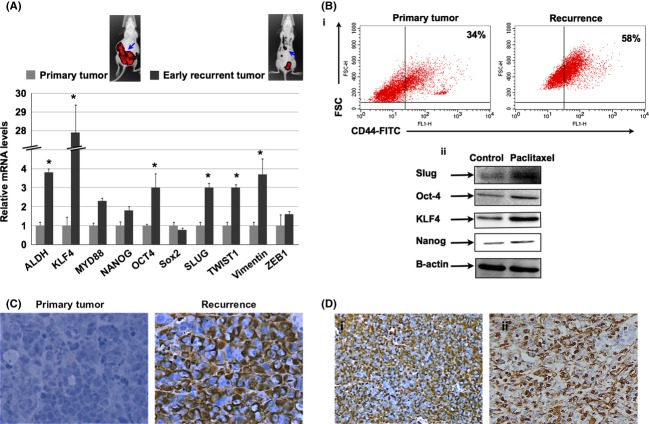
Differential gene expression pattern in Paclitaxel-surviving cells. (A) Differential mRNA expression levels of stemness- and mesenchymal-associated genes between primary tumors (not exposed to Paclitaxel) and recurrent tumors (following Paclitaxel). *n* = 10 animals per group; blue arrows point to tumors analyzed. (B-i) Flow cytometry analysis comparing CD44 levels in primary and recurrent tumors. Note the enrichment of CD44+ cells in recurrent tumors. (ii) Differential levels of protein expression in primary and recurrent tumors determined by Western blot analysis. Data shown are representative of three independent experiments (*n* = 10 animals per group). (C) Vimentin expression was compared between Control/primary tumors and Treated/recurrent tumors using immunohistochemistry. Data shown are representative of three independent experiments (*n* = 10 animals per group). (D) High levels of vimentin were also observed in samples from recurrent epithelial ovarian cancer (EOC) patients. Images are representative of immunohistochemistry staining from two patients. *P < 0.001.

As Cisplatin was not effective in reducing tumor burden and as its toxicity limited further dosing, we focused on analyzing the effect of Paclitaxel treatment on the tumors' molecular phenotype. Analysis of the gene signature of recurrent tumors (postpaclitaxel) showed that similar to that observed in vitro, recurrent disease is molecularly distinct from control no treatment/primary disease. Interestingly, we saw the same gene profile in vivo as observed in vitro. Compared to tumors from mice that were not exposed to Paclitaxel treatment (primary tumor), tumors from recurrent disease after treatment with Paclitaxel (early recurrent tumor) had higher levels of genes associated with stemness such as *ALDH1, KLF4, MYD88, NANOG,* and *OCT-4* (Fig. 5A). More importantly, we also observed higher levels of mesenchymal markers, *SNAI2* (*Slug*), *TWIST1*, and *VIM* in recurrent tumors (Fig. 5A). Upregulation of some of these markers were confirmed at the protein levels by flow cytometry or Western blot analysis and showed that Paclitaxel treatment enriched for CD44+ cells (Fig. 5B-i) and upregulated the expression levels of SLUG, OCT-4, KLF4, and NANOG in vivo (Fig. 5B-ii).

Histological analysis of the mouse tumors revealed morphological differences between the primary and recurrent tumors. Primary tumors display a more epithelial phenotype while recurrent tumors contain cells with mesenchymal-like morphology (Fig. S1). These morphological changes resemble those observed in ovarian cancer patients when primary disease is compared to recurrent disease (Fig. S1). Immunohistochemistry analysis for Vimentin further demonstrated the parallelism between mouse and human tumors. In mouse tumors, recurrent disease display very intense staining for the mesenchymal marker, Vimentin, compared to primary tumor (Fig. 5C). Analysis of recurrent tumors from patients with serous EOC likewise show higher expression levels of Vimentin in recurrent tumors compared to primary tumors (Fig. 5D). Taken together these results demonstrate the phenotypic modification in surviving CSC as a consequence of chemotherapy.

### SLUG overexpression in EOC cells induces a mesenchymal phenotype

**Figure 6:**
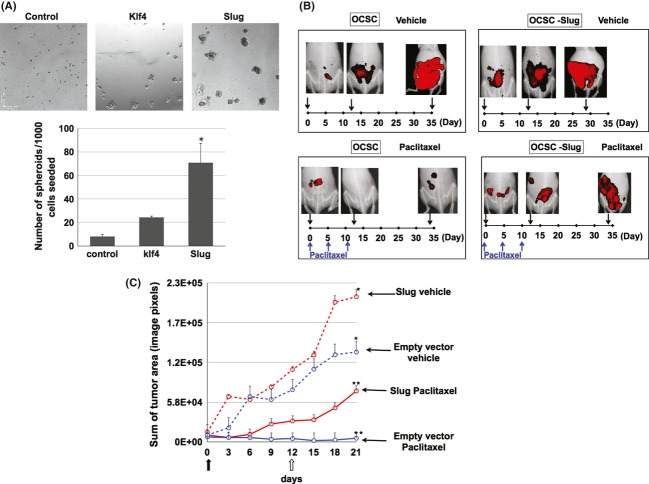
Slug promotes chemoresistance and aggressiveness. (A) Slug or Klf4 were overexpressed in ovarian cancer stem cell (OCSC1) and spheroid formation was quantified by manual counting. (B) Cells overexpressing Slug generated a more aggressive tumor in nude mice which did not respond to Paclitaxel. Images are representative of three independent experiments (*n* = 10 animals per group). (C) Tumor kinetics comparing vehicle-treated or Paclitaxel-treated mice harboring tumors established from OCSC1 transfected with empty vector or overexpressing Slug (Slug). 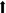 day 0 designated as beginning of treatment; 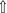 day 12 is the fourth and final dose of Paclitaxel. *P < 0.05 compared to Control. **, *P < 0.001 compared to control no treatment (*n* = 10 animals per group).

As we observed both in vitro and in vivo that OCSC1 that survived Paclitaxel have elevated levels of SLUG and KLF4, we next determined the functional significance of upregulation of these proteins. Thus, we stably transfected OCSC1 with either SLUG or KLF4. Our results show that overexpression of SLUG and KLF4 enhanced spheroid formation in OCSC1 compared to empty plasmid. Interestingly, SLUG overexpression had a more pronounced effect on spheroid forming capacity than KLF4 (Fig. 6A). These results suggest that while maintaining their stemness potential, the CSC that survived Paclitaxel acquired anchorage-independent growth characteristics and migratory potential.

### SLUG overexpression accelerates tumor growth and induces resistance to Paclitaxel

Finally, we determined whether SLUG overexpression affects the response to Paclitaxel. Our results show that SLUG overexpression can induce Paclitaxel resistance (Fig. S2) Therefore, we tested the effect of SLUG overexpression in our animal model. When tested in vivo, xenografts generated from mCherry+ OCSC1-F2 cells overexpressing SLUG were resistant to Paclitaxel (Fig. 6B) and tumor progression was significantly enhanced. More importantly, analysis of the tumor kinetics in the nontreated control groups showed that SLUG overexpression resulted in tumors with faster growth kinetics and therefore a more aggressive form of tumor (Fig. 6C). That was not the case for OCSC transfected with KLF4 (data not shown). Taken together these results suggest that when SLUG is induced in response to Paclitaxel, these cells acquire a more aggressive and resistant phenotype.

## Discussion

In the present study, we demonstrate that CD44+/MyD88+ EOC stem cells are inherently resistant and can survive Paclitaxel treatment. Using unique in vitro and in vivo models, which resemble the clinical profile of recurrence in patients with EOC, we demonstrate that Paclitaxel treatment can induce molecular modifications on the preexisting CSC, enhancing the acquisition of mesenchymal characteristics, while maintaining its stemness potential. Our findings suggest that chemotherapy does not only enrich for putative EOC stem cells, but also that treatment can also induce specific phenotypic modifications in the surviving EOC stem cells.

EOC is the gynecologic malignancy with the highest case to mortality ratio [2]. Mortality in EOC is almost always associated with recurrent disease. Newly diagnosed patients exhibit initial responsiveness to treatment but eventually recur. It is estimated that 25% of patients recur within 6 months and are therefore considered chemoresistant [34]. Indeed, in this patients who are considered platinum resistant, second round chemotherapy gives only 5–10% response rate [34]. This shows that the therapy that was effective against primary disease fails to induce a response in recurrent disease and suggests that primary disease may be distinct from recurrent disease. The data presented in this study provide experimental evidence of these phenotypic differences and demonstrate specific molecular changes that occur during the process of disease recurrence.

Inherent chemoresistance has been ascribed to CSC [35]. This can be viewed as the first step for disease recurrence. Our group, as well as others, has shown that recurrent EOC is more densely populated by CD44+ EOC stem cells [18, 28, 36, 37]. This suggests that CSC survive chemotherapy. Indeed, results presented in this study and in our group's previous publications and other groups' reports, have highlighted the inherent chemoresistance of CD44+ EOC cells [8, 22–24]. Moreover, it has been reported that the percentage of ovarian CSC identified as a side population is enhanced by Paclitaxel [38]. It can be stated that the second step in the process of recurrence is the rebuilding of the tumor. Thus, a second implication of the enrichment of CSC in recurrent disease is that instead of undergoing differentiation to rebuild the tumor, CSC activates self-renewal pathways that maintain stemness-associated characteristics. We show in this study that the surviving cells (after Paclitaxel treatment) maintain key EOC stemness-associated genes such as CD44, ALDH1, MyD88, NANOG, OCT4, and SOX2. This supports our previous finding that tumor injury induced either by surgery or chemotherapy promotes the process of self-renewal in these cells [9]. It also parallels previous studies that showed upregulation of CD44, ALDH1, and CD133 in recurrent human EOC samples compared to their matched primary tumors [36].

An important finding in this study is the observation that in the EOC stem cells that survive Paclitaxel, in addition to the activation of self-renewal pathways, we also observe upregulation of genes associated with EMT (SLUG, KLF4, Twist1, and Vimentin). These molecular changes might provide the original epithelial CSC the capacity to migrate and establish multiple metastatic sites, a characteristic of recurrent disease.

The in vivo i.p. recurrence model described in this study mimics the clinical profile of recurrent disease observed in EOC patients. A limitation is that in our model, the use of combination chemotherapy (platinum plus taxane), which is usually given to patients, is toxic. However, it is interesting to note that prolonged treatment with low-dose Cisplatin likewise results in upregulation of stemness markers as well as EMT in ovarian cancer cell lines in vitro [39].

Our results show that SLUG is one of the more significantly upregulated genes in Paclitaxel-surviving CSC. Interestingly, SLUG overexpression in OCSC1 is sufficient to confer anchorage-independent growth in vitro. More importantly, SLUG overexpression is sufficient to yield a more aggressive and more chemoresistant tumors. SLUG is a transcription factor encoded by the gene *SNAI2* and belongs to the Snail family of C2H2-type zinc finger transcription factors [40, 41]. Together with other transcriptions factors known to induce EMT (i.e., Twist and Zeb), a main function that has been attributed to SLUG is the repression of genes associated with the epithelial phenotype. These include genes that encode for cadherins, claudins, and cytokeratins [35]. SNAIL and SLUG have also been associated with chemoresistance and activation of stemness-associated pathways [42, 43]. It is therefore not surprising that ectopic expression of SLUG in OCSC1 was sufficient to promote a more aggressive and chemoresistant phenotype. The significance of our findings is the possibility of developing new venues to target SLUG as part of maintenance therapy and therefore prevent recurrence and metastasis, the main causes of mortality in patients with ovarian cancer.

**Figure 7:**
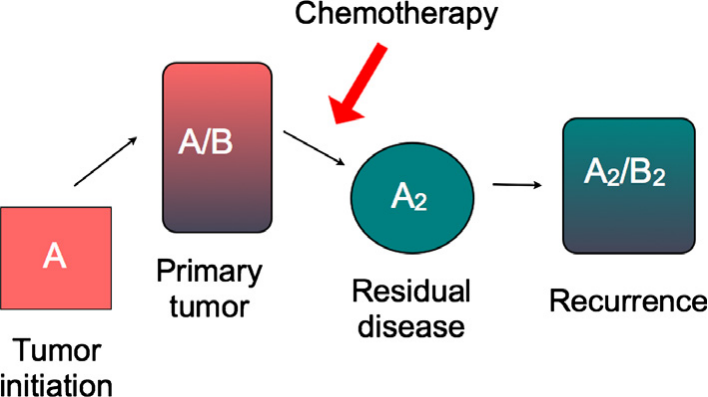
Depiction of proposed modifications within the tumor during chemotherapy and recurrence. Primary tumor is heterogeneous and composed of at least two types of cancer cells: the inherently chemoresistant tumor-initiating cells, A, and the chemosensitive-differentiated cancer cells, B. Chemotherapy will induce cell death in B but not in A. Due to its plasticity, surviving A undergoes molecular modification to A2, which then differentiates into B2 to rebuild the tumor.

In conclusion, we demonstrate using in vitro and in vivo models that putative ovarian CSC, which survive chemotherapy can acquire molecular phenotypic modifications that makes them distinct from the original tumor-initiating cells (Fig. 7). Cancer cells exhibit dynamic phenotypic changes during the process of tumor progression. These changes may be significantly influenced by treatment modalities. After chemotherapy, the cancer population that survive and rebuild the tumor will be phenotypically different from the tumor-initiating cells. The modifications that occur may not be the same in every patient. This suggests that treatment modalities should be modified to each individual patient. Further studies using our models will identify biomarkers for personalized treatment.

## Supporting Information

**Figure S1.** Primary and recurrent EOC tumors obtained from mouse xenograft are morphologically distinct and parallels the difference observed in patient samples.

**Figure S2.** OCSC1 overexpressing GFP or Slug were treated with Paclitaxel. Note that overexpression of Slug yields more resistant cultures.
