# Phenotypic modifications in ovarian cancer stem cells following Paclitaxel treatment

**DOI:** 10.1002/cam4.165

**Published:** 2013-12-02

**Authors:** Vinicius Craveiro, Yang Yang-Hartwich, Jennie C Holmberg, Natalia J Sumi, John Pizzonia, Brian Griffin, Sabrina K Gill, Dan-Arin Silasi, Masoud Azodi, Thomas Rutherford, Ayesha B Alvero, Gil Mor

*Cancer Medicine* 2013; **2**: 751–762

The name of Dr. Won Duk Joo was inadvertently omitted from the list of authors. The author list should read:

Vinicius Craveiro, Yang Yang-Hartwich, Jennie C. Holmberg, Won Duk Joo, Natalia J. Sumi, John Pizzonia, Brian Griffin, Sabrina K. Gill, Dan-Arin Silasi, Masoud Azodi, Thomas Rutherford, Ayesha B. Alvero & Gil Mor

The corresponding author apologises for this error. The affiliation for Dr. Joo is:

Department of Obstetrics, Gynecology and Reproductive Sciences, Reproductive Immunology Unit, Yale University School of Medicine, New Haven, Connecticut; and Department of Obstetrics and Gynecology, CHA Gangnam Medical Center, CHA University, Seoul, Republic of Korea
