# Supplementary material for: Phenotypic modifications in ovarian cancer stem cells following Paclitaxel treatment
Source: Cancer Med. 2013 Aug 27;2(6):751–62. doi: 10.1002/cam4.115 (PMC3892380; doi:10.1002/cam4.115)
Supplement: Supplementary file 1 [file cam40002-0751-SD1.pdf]

Legends to Supplementary Figures

Supplementary Fig. 1. Primary and recurrent EOC tumors obtained from mouse xenograft are morphologically distinct and parallels the difference observed in patient samples.

Supplementary Fig. 2. OCSC1 overexpressing GFP or Slug were treated with Paclitaxel. Note that overexpression of Slug yields more resistant cultures.

Supp. Fig. 1

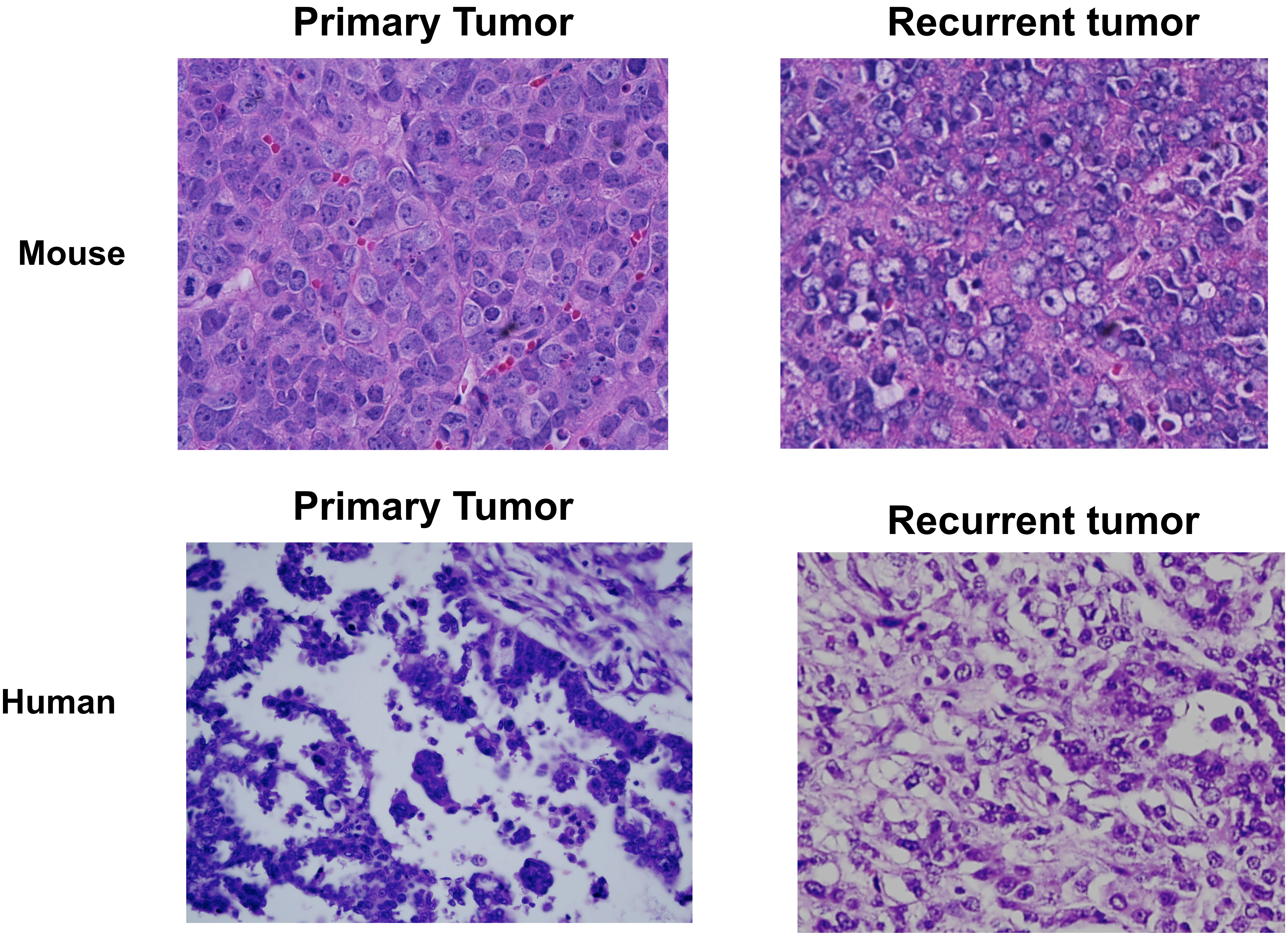
Primary Tumor
Recurrent tumor
Mouse
Primary Tumor
Recurrent tumor
Human

Supp. Fig. 2

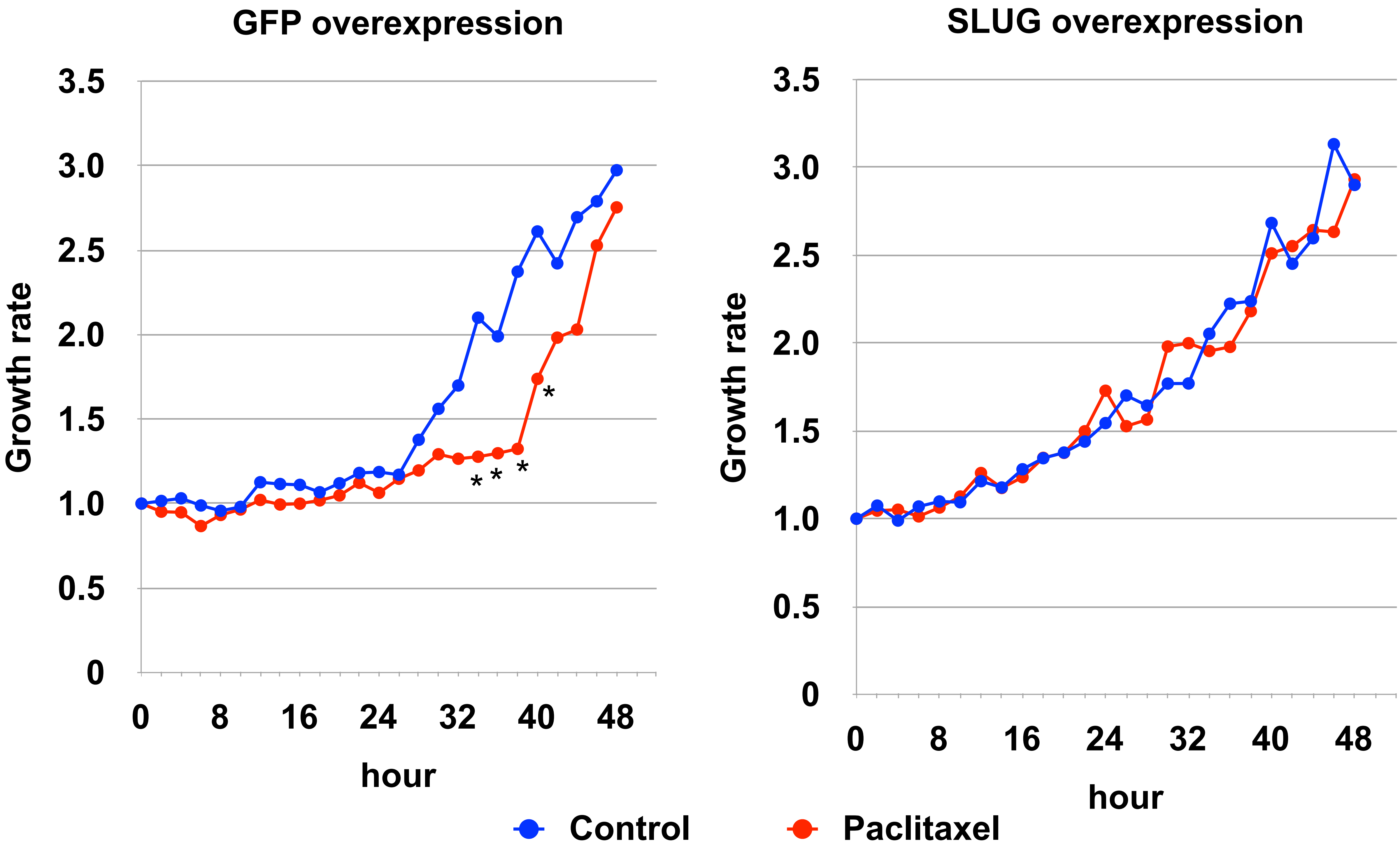
GFP overexpression
SLUG overexpression
Growth rate
3.5
3.0
2.5
2.0
1.5
1.0
0.5
0
0
8
16
24
32
40
48
hour
*
*
*
*
Control
Paclitaxel
